# Ultrafast Parallel Micro-Gap Resistance Welding of an AuNi_9_ Microwire and Au Microlayer

**DOI:** 10.3390/mi12010051

**Published:** 2021-01-03

**Authors:** He Zhang, Shang Wang, Bingying Wu, Weiwei Zhang, Chunjin Hang, Yanhong Tian

**Affiliations:** 1 State Key Laboratory of Advanced Welding and Joining, Harbin Institute of Technology, Harbin 150001, China; 19B909123@stu.hit.edu.cn (H.Z.); wangshang@hit.edu.cn (S.W.); 18245020417@163.com (B.W.); hangcj@hit.edu.cn (C.H.); 2 Institute of Electronic Engineering, China Academy of Engineering Physics, Mianyang 621900, China; zhangweiwei0509103@163.com

**Keywords:** AuNi_9_ microwire, Au microlayer, parallel micro-gap resistance welding

## Abstract

Welding between an AuNi_9_ microwire and Au microlayer is of great importance for fabricating electrical contact structures for high precision inertial devices, satellite slip ring brushes, robots, etc. In this paper, the achievement of parallel micro-gap resistance welding (PMRW) with 200-μm AuNi_9_ microwires on a 3-μm Au layer was presented. The study on the orthogonal design of the experiment was carried out. The effect of the process parameters (welding current, welding time, and welding pressure) was discussed in reference to the morphologies and tensile force of the joint using range analysis. It is shown that too much or too little heat input will decrease the welding performance. A group of optimized process parameters (0.275 kA welding current, 3 ms welding time, and 28.7 N welding pressure) was obtained. During the welding process, the dynamic resistance of the whole welding system was measured, which can reflect the welding quality. Finite element simulation is utilized to calculate the welding temperature. The highest temperature was located in the center area of the AuNi_9_ microwire, reaching 1397.2 °C, which is higher than the melting point of AuNi_9_. By contrast, the highest temperature for the pad was 826.47 °C (lower than the melting point of Au). Hence, under optimized process parameters, a transient interfacial reaction between the liquid AuNi_9_ microwire and solid Au pad occurred, and the strength of the welded joint reached 5.54 N.

## 1. Introduction

As a result of its excellent electrical conductivity, chemical stability, and corrosion resistance, gold is widely used as an electronic sliding contact material for high precision inertial devices, satellite slip ring brushes, robots, etc. [1,2,3,4,5]. However, the low intrinsic hardness, easy deformation, and poor elasticity of pure gold limits its application in circumstances necessitating high mechanical strength requirements [6,7]. In order to improve the mechanical performance, many strengthening elements, including yttrium, cobalt, and nickel, are added [7,8,9]. The AuNi_9_ alloy is considered to be one of the most promising materials because of its high strength, low resistivity, and stability. In such applications, AuNi_9_ brushes are usually assembled with Au sheets so as to fabricate friction pairs. Hence, a robust and reliable welding joint between a microscale AuNi_9_ wire and Au layer is highly required.

Tin-based solder is commonly used for welding AuNi_9_ microwires and Au microlayers. However, brittle intermetallic compounds are easily formed between gold, nickel, and tin, such as AuSn_4_, Ni_3_Sn_4_, and AuNi_2_Sn_4_, which have a tremendous negative effect on the mechanical performance of joints [10,11,12,13]. Indium-based solder was developed to suppress the formation of intermetallic compounds, but the increased cost hinders its large-scale application [14]. Furthermore, because the soldering method needs added flux, solder, and heat for a long time (generally >10 s), the process is complicated and time-consuming. Hence, a fast, facile, and cost-effective welding method is urgently needed to weld AuNi_9_ and Au. Thanks to its simple process and high efficiency, parallel micro-gap resistance welding (PMRW) seems to be a good alternative, which uses the heat generated by the electric current flow to weld the connected materials [15,16,17]. Moreover, as no solder is added during the welding process, the formation of brittle intermetallic compounds is effectively avoided. Over the past few decades, massive studies have been carried out regarding the welding of microscale wires and sheets utilizing the PMRW method; 40-μm copper microwires have successfully been welded onto a gold layer using this method and resulting in a high strength. The welding interface resistance showed no change during the random vibration process, indicating is excellent reliability [18,19,20]. Firm joining between a 150-μm nickel wire and nichrome layer was achieved by Wang et al., who utilized PMRW [21], and the effect of the welding pressure was discussed. These results proved the feasibility of welding AuNi_9_ microwires and Au microlayers using this method. However, systematic research on the welding of these two materials is still lacking.

In this study, we successfully welded an AuNi_9_ microwire and Au microlayer using the ultrafast (less than 6 ms) PMRW method. Compared with the conventional soldering method, the welding time decreased by two orders of magnitude. The process parameters (current, time, and pressure) were systematically investigated through orthogonal experiments, and the influence degree of these three factors on the strength was compared. Furthermore, we in-situ monitored the dynamic resistance during the welding process to characterize the welding quality. Based on the finite element simulation, the welding mechanism was discussed.

## 2. Materials and Methods

**Figure 1:**
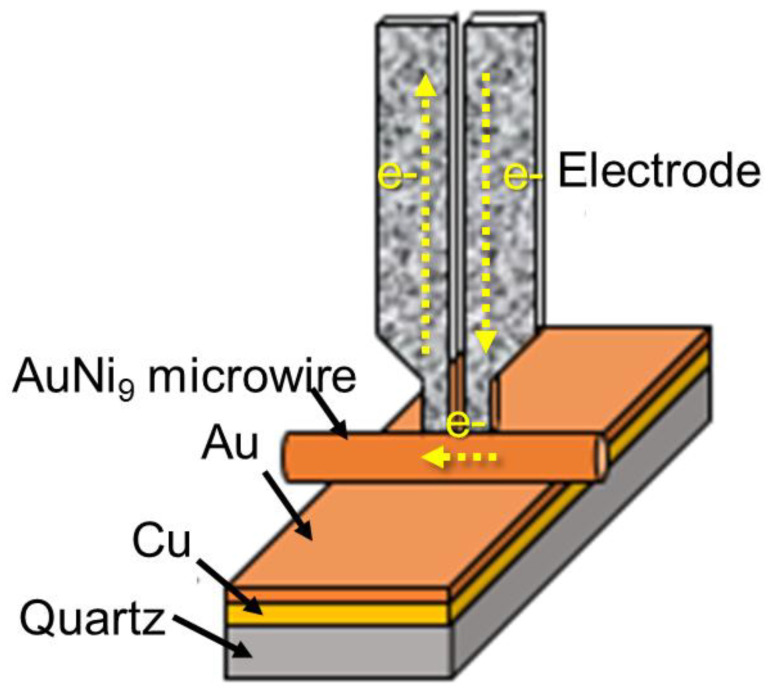
The welding installation and welding structure of an AuNi_9_ microwire and Au microlayer.

**Figure 2:**
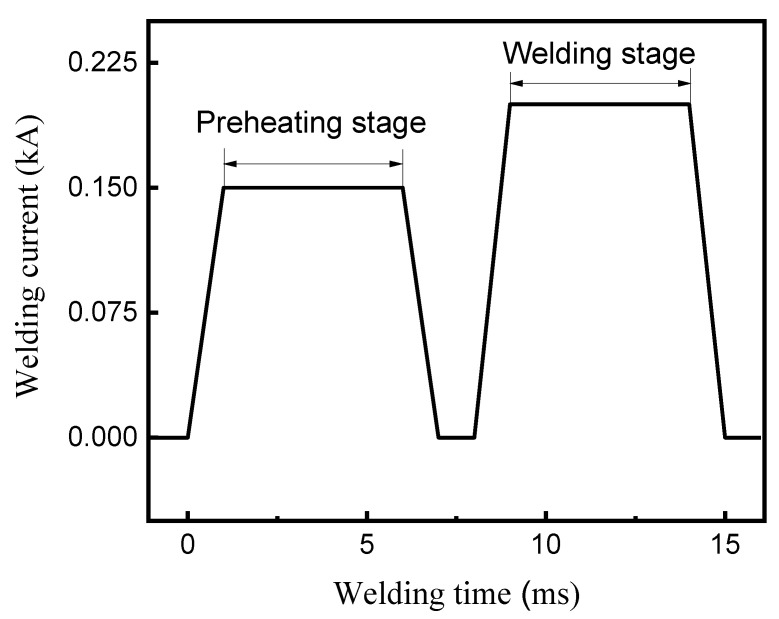
A typical welding current waveform.

Figure 1 shows the welding installation and welding structure of the specimen used in the experiment. The diameter of the AuNi_9_ microwire was 200 μm. The substrate consisted of three layers—the top gold layer (3 μm), the middle copper layer (10 μm), and the bottom quartz layer. In the general welding process, the AuNi_9_ wire and substrate were first cleaned sequentially with acetone and absolute ethanol for 30 s to remove surface contaminants using an ultrasonic cleaner. Then, an AuNi_9_ microwire was placed onto the substrate. The welding experiment was achieved through a micro-resistance spot welding resistance system (Unitek, Monrovia, CA, USA), and the output current waveform was the double pulse output mode. During the pre-heat stage, the contact surface was softened and became smoother. It is worth pointing out that all of the parameters mentioned in the following were all in the welding stage. The parameters of the pre-heating stage were fixed at 150 A-5 ms. Figure 2 exhibits a typical welding current waveform.

**Figure 3:**
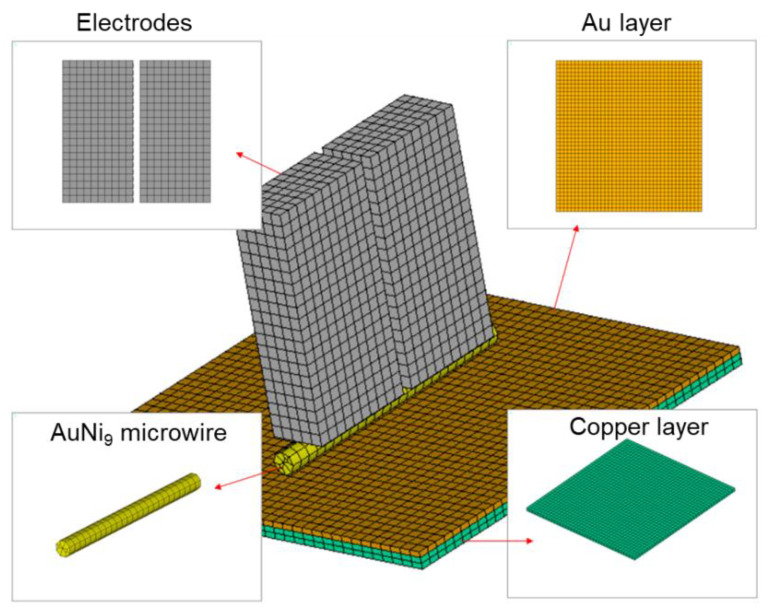
Finite element model of the welding structure.

In order to better understand the welding mechanism between an AnNi_9_ microwire and Au microlayer, a finite element model was built to simulate the welding process using the software ANSYS. Figure 3 shows the finite element model. The direct coupling method was selected in order to improve the simulating accuracy using the interaction of electric-thermal–structural fields during the parallel resistance welding process. The Solid226 coupling element was utilized to describe the microwire and the substrate, and contact elements Target170 and Contact174 were employed to simulate the welding contact and disconnection. The size of the finite element model was the same as the actual welding structure. 

**Figure 4:**
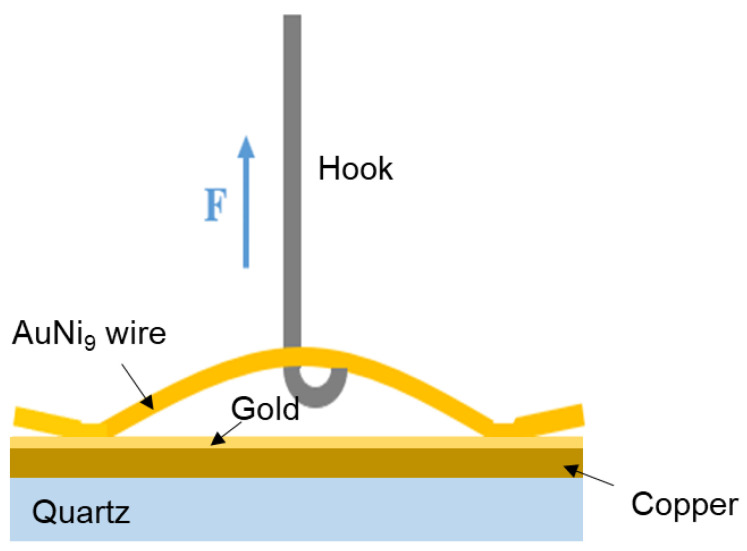
Schematic diagram of measuring the tensile force.

The morphologies of the welding structure under different parameters were characterized by scanning electron microscopy (SEM; FEI, Hillsboro, OR, USA). Element analysis was achieved using an energy dispersive spectrometer (EDS) equipped with SEM. The feedback voltage was measured in-situ to obtain the dynamic resistance during the welding process, the voltage was measured in-situ using storage by employing the data recorder (HIOKI, Ueda, Japan). Then, the dynamic real-time resistance could be calculated using Ohm’s law. Figure 4 schematically illustrates the measurement of the welded joint strength. In this work, the welded joint strength was evaluated using tensile force, which was measured via a universal testing machine (Instron, Boston, MA, USA).

## 3. Results and Discussion

**Figure 5:**
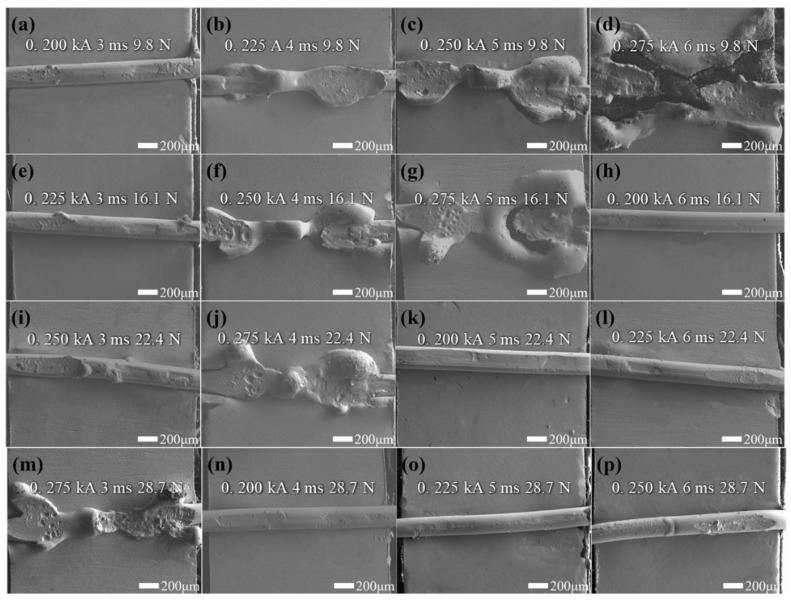
View of welded joints (**a**–**p**) based on the parameters a–p in Table 1.

The welded joint strength between the AnNi_9_ microwire and Au microlayer is the most important index, which mainly depends on the Joule heat and pressure during the welding process. Moreover, the Joule heat can be adjusted using the welding current and welding time. Hence, we systematically studied the effect of these three parameters on the welded joint strength. According to previous tests, the ranges of the welding parameters were initially determined as follows: 0.2–0.275 kA welding current, 3–6 ms welding time, and 9.8–28.7 N welding force. To obtain the optimized welding parameters, an orthogonal experiment (three factors with four levels) was designed. The detailed experimental parameters and results are shown in Table 1, and the corresponding morphologies under different welding parameters are sho0wn in Figure 5.

Figure 5a–d shows the morphologies at a fixed pressure of 9.8 N. When inputting a current of 0.200 kA for 3 ms, the AuNi_9_ microwire deformed slightly and no apparent melting was observed (Figure 5a). At this time, the welded joint was poor. As the welding parameters (welding current and welding time) increased, the AuNi_9_ microwires deformed increasingly. The welding quality was improved and a higher tensile force was measured. However, when the welding current increased to 0.275 kA and the welding time increased to 6 ms, the temperature was so high that the plated layer was destroyed and the substrate was exposed (Figure 5d). Both the AnNi_9_ microwire and Au microlayer were obviously melted. These results can easily be explained by the increasing generation of Joule heat, according to Joule’s law (Q = I^2^RT). With the increase of in the welding current and welding time, more heat would generate at the center area, promoting joining between the AuNi_9_ microwire and Au microlayer. Nevertheless, when the welding parameters were too great, the excessive temperature would damage the pad and the welded joint strength would decrease. The welding pressure had two effects on the welding morphology. On the one hand, a large pressure will increase the deformation of the microwire. On the other hand, under a larger welding pressure, the contact area between the AuNi_9_ wire and Au layer will increase, resulting in a decrease in the electrical resistance, and the Joule heat would decrease. For example, as shown in Figure 5b,p, although larger welding parameters are applied in Figure 5p, more obvious deformation is observed in Figure 5b. Based on the above analysis, the welding current and welding time directly affect the amount of joule heat generation. Although the effect of welding pressure cannot intuitively be observed through Joule’s law, it can affect the generation of Joule heat by changing the contact resistance. Hence, its necessary to comprehensively analyze the effect of the parameters on the welded joint strength.

Orthogonal experiments can obtain the optimized process parameters through the least experiments, and can ensure the primary and secondary factors. It can be found from Table 1 that when the welding current is 0.275 kA, the welding time is 3 ms, and the welding pressure is 28.7 N; here, the greatest welded joint strength was obtained. According to the range analysis results in Table 2, the order of the significance of these three factors is as follows: welding current > welding time > welding force. The results show that welding current has the most significant impact on welded joint strength, which is attributed to the quadratic relationship between current and Joule heat.

**Figure 6:**
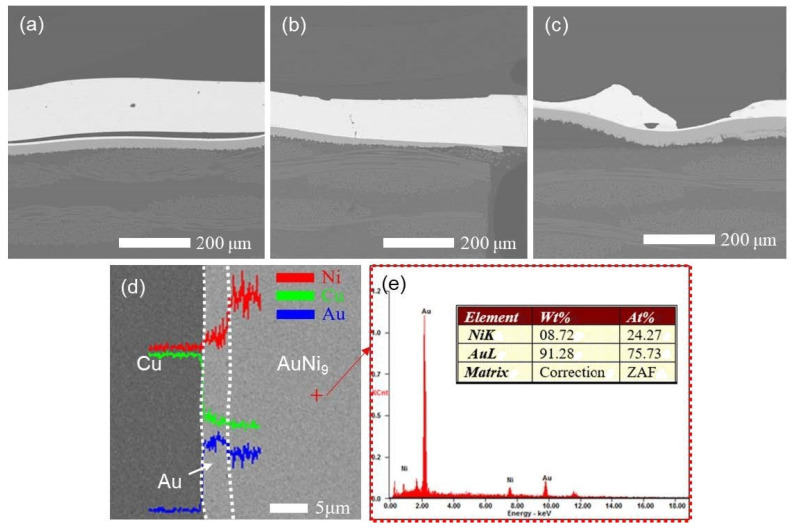
Cross-sectional SEM images of welding interface between AuNi_9_ microwire and Au microlayer at (**a**) 0.200 kA, 3 ms, and 9.8 N; (**b**) 0.275 kA, 3ms, and 28.7 N; and (**c**) 0.275 kA, 6 ms, and 9.8 N. (**d**) Elemental line scan of the welding interface at the optimized parameters. (**e**) Element composition of the microwire. The parameters correspond with designations a, m, and d in Table 1, respectively.

The cross-sectional interfaces of the AuNi_9_ microwire and Au microlayer at three typical parameters were characterized using SEM in the backscattered electrons mode, as depicted in Figure 6a. When the output of heat was too low at small parameters (0.200 kA, 3 ms, and 9.8 N), an obvious gap between them was observed, which is consistent with poor welded joint strength. As the parameters increased, the joining interface between the AuNi_9_ microwire and Au microlayer was dense and void-free, indicating the formation of a robust metallurgical joint. When the Joule heat was too high, overmelting of the microwires was also observed for the cross-sectional view. Figure 6b shows the results of the elemental line scanning across the welding interface from the substrate to the microwire. The elements Au, Ni, and Cu were continuously distributed along the welding interface. Furthermore, the composition of the microwire was also analyzed by EDS. As shown in Figure 6c, the chemical composition of the microwire was Au 91 wt % and Ni 9 wt %, proving the existence of AuNi_9_.

**Figure 7:**
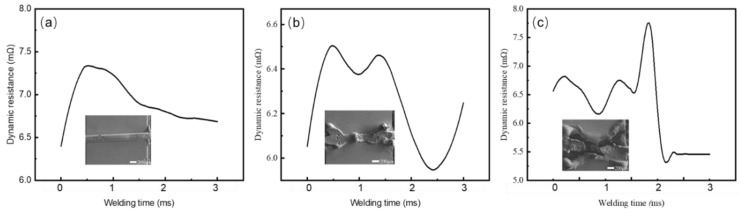
Dynamic resistance curves during the welding process at (**a**) 0.200 kA, 3 ms, and 9.8 N; (**b**) 0.275 kA, 3 ms, and 28.7 N; and (**c**) 0.275 kA, 6 ms, and 9.8 N. The inset images are the corresponding morphologies. The parameters correspond with designations a, m, and d in Table 1, respectively.

Many researchers have reported that the resistance of joints can reflect the welding quality [22,23,24]. This method is non-destructive and can be monitored in real time. Hence, resistance during the welding PMRW process was measured in-situ to evaluate the welding quality of the AuNi_9_ microwire and Au microlayer. We tested the dynamic resistance under three typical process parameters during the welding process, as shown in Figure 7. When the parameters were small (0.200 kA, 3 ms, and 9.8 N), the heat generated was not enough to cause the materials to melt. At this time, the AuNi_9_ microwires and Au layers were only slightly softened, and only one peak was observed in the dynamic resistance curve (Figure 7a) [25,26]. With the increase in parameters, a second peak was observed, indicating the formation of the nugget. The resistance dropped sufficiently after the second peak, which revealed the growth of the nugget (Figure 7b) [27]. However, when too much Joule heat was generated, the resistance dropped rapidly (Figure 7c), and excessive melting of the connected material was observed, as shown in the inset image of Figure 7c.

**Figure 8:**
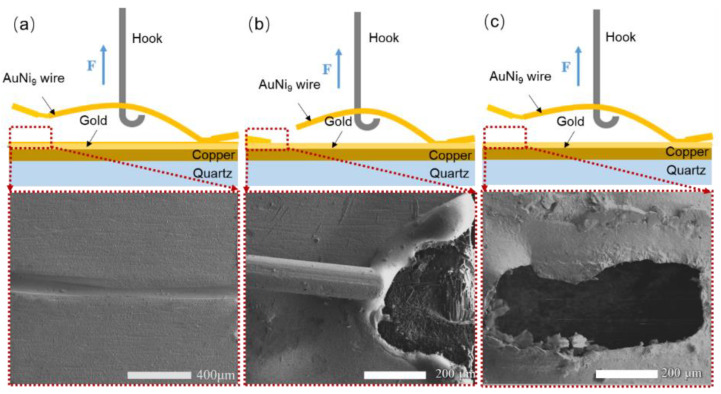
Schematic illustrations and corresponding fracture morphologies at (**a**) 0.200 kA, 3 ms, and 9.8 N; (**b**) 0.275 kA, 3 ms, and 28.7 N; and (**c**) 0.275 kA, 6 ms, and 9.8 N. The parameters correspond with the designations a, m, and d in Table 1, respectively.

Figure 8 shows the representative fracture morphologies of the welded joints under the afore-mentioned three typical parameters. When the generated Joule heat was low, a poor joint between the AuNi_9_ microwire and Au layer was obtained, and the AuNi_9_ microwire was easily peeled off from the pad, as shown in Figure 8a,d. When the parameters increased, the joint was fractured at the neck, indicating the strength of the welded joint was more robust than that of the AuNi_9_ wire. As the generated Joule heat increased further, the entire joint was detached from the quartz surface, caused by damage to the Au microlayer during the welding process.

**Figure 9:**
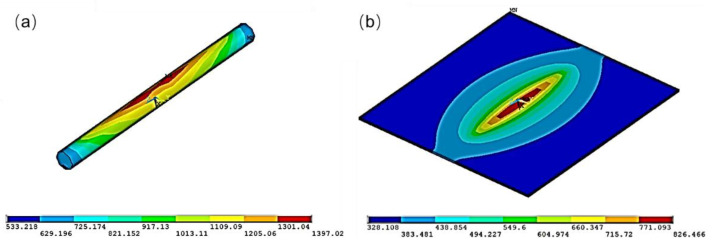
Temperature distribution of (**a**) an AuNi_9_ microwire and (**b**) Au layer under the optimized parameters (0.275 kA, 3 ms, and 28.7 N).

**Figure 10:**
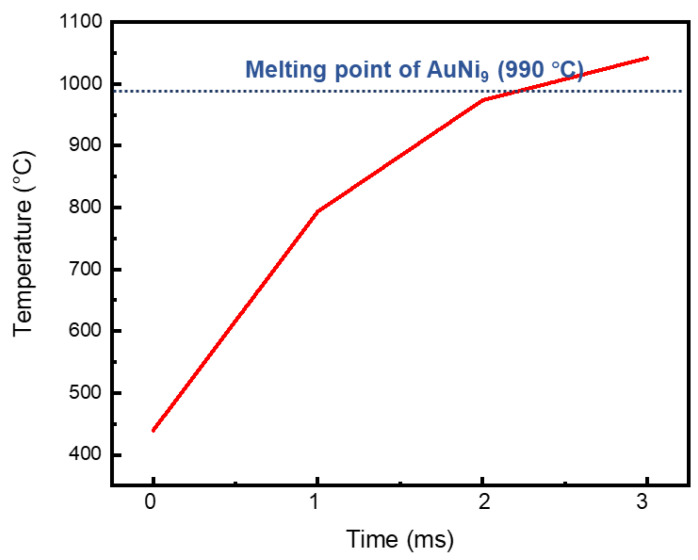
Temperature curve of an AuNi_9_ microwire under the optimized parameters (0.275 kA, 3 ms, and 28.7 N).

With the help of finite element simulation, we calculated the temperature distribution at the optimized parameters during the welding process, as shown in Figure 9. Regardless of the microwire or the microlayer, the high temperature was concentrated on the center area (red color area), where the electric current mainly flows. The temperature of the AuNi_9_ microwire was higher than that of the Au layer. The highest temperature on the AuNi_9_ microwire reached 1397.02 °C, which is higher than the melting point of AuNi_9_ (990 °C). By contrast, the highest temperature for the Au layer was only 826.47 °C, which is far lower than the melting point of Au (1064.18 °C). Hence, during the ultrafast welding process, the AuNi_9_ microwire was melted and the Au pad remained in a solid state at the interface. A transient interfacial reaction between liquid AuNi9 and solid Au occurred. Sufficient elemental interdiffusion was also observed (as indicated in Figure 6d), forming a robust metallic joint. The average temperature during the welding process is shown in Figure 10. When applying the parameters of 0.275 kA, 3ms, and 28.7 N, the average temperature raised, reaching the melting point of AuNi_9_. During the cooling process, the melted area of the AuNi_9_ microwire re-solidified, and a robust welding joint was obtained.

## 4. Summary

In summary, we successfully welded an AuNi_9_ microwire and Au microlayer using the facile and ultrafast PMRW method. Orthogonal experiments were carried out to optimize the process parameters.

(1) Here, 0.275 kA welding current, 3 ms welding time, and 28.7 N welding pressure were the optimal parameters for achieving the highest tensile force. Too much or too little Joule heat input will reduce the welded joint strength. 

(2) The range analysis showed that the welding current is the main factor, and welding time and welding force are the secondary factors. 

(3) We calculated the welding temperature using the finite element simulation method. During the welding process, the temperature of the AuNi_9_ microwire was higher than its melting point, so it will melt. After re-solidification during the cooling process, a robust welded joint with a gold layer was obtained.

## Figures and Tables

**Table 1:** Orthogonal experiment parameters and tensile force results.

Designation	Welding Current (kA)	Welding Time (ms)	Welding Pressure (N)	Tensile Force (N)
a	0.200	3	9.8	0.14
b	0.225	4	9.8	0.37
c	0.250	5	9.8	2.33
d	0.275	6	9.8	0.46
e	0.225	3	16.1	1.05
f	0.250	4	16.1	1.62
g	0.275	5	16.1	0.84
h	0.200	6	16.1	0.18
i	0.250	3	22.4	0.58
j	0.275	4	22.4	3.36
k	0.200	5	22.4	0.20
l	0.225	6	22.4	0.87
m	0.275	3	28.7	5.54
n	0.200	4	28.7	0.62
o	0.225	5	28.7	0.23
p	0.250	6	28.7	1.15

**Table 2:** Range analysis of the orthogonal experiments.

	Factor	Welding Current	Welding Time	Welding Pressure
Average Tensile Force (N)				
At level 1		0.29	1.83	0.83
At level 2		0.63	1.49	0.92
At level3		1.42	0.90	1.25
At level 4		2.25	0.67	1.89
Range		1.96	1.16	1.06
